# Ultrasound Hypotension Protocol Time-motion Study Using the Multifrequency Single Transducer Versus a Multiple Transducer Ultrasound Device

**DOI:** 10.5811/westjem.2020.12.47862

**Published:** 2021-04-08

**Authors:** Linda Sabbadini, Rocco Germano, Emily Hopkins, Jason S. Haukoos, John L. Kendall

**Affiliations:** * University of Brescia - Medicine and Surgery, Department of Clinical and Experimental Science, Brescia, Italy; † Denver Health Medical Center, Department of Emergency Medicine, Denver, Colorado; ‡ University of Colorado School of Medicine, Aurora, Colorado; § Colorado School of Public Health, Aurora, Colorado

## Abstract

**Introduction:**

Ultrasound hypotension protocols (UHP) involve imaging multiple body areas, each with different transducers and imaging presets. The time for task switching between presets and transducers to perform an UHP has not been previously studied. A novel hand-carried ultrasound (HCU) has been developed that uses a multifrequency single transducer to image areas of the body (lung, heart, abdomen, superficial) that would typically require three transducers using a traditional cart-based ultrasound (CBU) system. Our primary aim was to compare the time to complete UHPs with a single transducer HCU to a multiple transducer CBU.

**Methods:**

We performed a randomized, crossover feasibility trial in the emergency department of an urban, safety-net hospital. This was a convenience sample of non-hypotensive emergency department patients presenting during a two-month period of time. Ultrasound hypotension protocols were performed by emergency physicians (EP) on patients using the HCU and the CBU. The EPs collected UHP views in sequential order using the most appropriate transducer and preset for the area/organ to be imaged. Time to complete each view, time for task switching, total time to complete the examination, and image diagnostic quality were recorded.

**Results:**

A total of 29 patients were scanned by one of eight EPs. When comparing the HCU to the CBU, the median time to complete the UHP was 4.3 vs 8.5 minutes (P <0.0001), respectively. When the transport and plugin times were excluded, the median times were 4.1 vs 5.8 minutes (P <0.0001), respectively. There was no difference in the diagnostic quality of images obtained by the two devices.

**Conclusion:**

Ultrasound hypotension protocols were performed significantly faster using the single transducer HCU compared to a multiple transducer CBU with no difference in the number of images deemed to be diagnostic quality.

## INTRODUCTION

Shock is one of the most challenging diagnostic presentations for the emergency physician (EP), and it is associated with mortality that has been reported as high as 25%.^1^ Physical examination alone, in most instances, is insufficient to adequately determine the etiology of shock, due to the complexity of the pathophysiological mechanisms, which can result in delayed diagnosis and appropriate treatment.^2^ Outcomes of patients in shock are closely related to the duration of hypotension; therefore, it is crucial to reach the correct diagnosis as soon as possible in order to institute the most appropriate therapy.^3^

The rapid ultrasound for shock and hypotension (RUSH) examination is a scanning protocol that involves imaging the chest, heart, peritoneal cavity, abdominal aorta, inferior vena cava (IVC), and leg veins to determine the etiology of different shock states.^4^ Similar ultrasound hypotension protocols (UHP) have been shown to improve outcomes, enhance diagnostic certainty, and change patient management.^2^,^5^–^7^ For example, Haydar et al found that incorporating an UHP involving multiple views of the heart and IVC altered more than half of physicians’ management decisions, while 90% perceived the ultrasound data to have positive clinical utility.^5^ Similarly, Shokoohi et al used an UHP involving a focused cardiac assessment, measurement of IVC collapsibility, an assessment of the abdominal cavity for free fluid or abdominal aortic aneurysm, and a thoracic scan to evaluate for pneumothorax, which changed management in 24.6% of hypotensive patients while decreasing diagnostic complexity by 27.4%.^6^

A unique aspect of an UHP is the need to scan multiple different body regions and structures. While previous studies have recommended using specific transducers to perform different aspects of the UHP, such as a phased array for the cardiac component, curvilinear for the abdominal structures, and a linear for the lung and extremity venous examinations,^4^ the only study reporting time to complete a multiorgan hypotension protocol employed a single transducer.^2^ Consequently, it is unclear how much time is needed to complete an UHP using specific transducers and presets for the different components of the examination.

A novel hand-carried ultrasound (HCU) system, the Butterfly iQ, (Butterfly Network, Inc., Guilford, CT) uses a single, multifrequency, capacitive micromachined ultrasonic transducer (CMUT) (1–9 megahertz [MHz]) to image areas of the body (lung, heart, abdomen, superficial) that would typically require three piezoelectric transducers using a traditional cart-based ultrasound (CBU) system. A potential advantage of this design is decreasing the need for task switching to select different presets and transducers, which may result in less time to perform multiorgan ultrasound protocols and improve efficiency for physicians in the emergency department (ED). The primary aim of our study was to measure the time needed to perform an UHP with a single transducer HCU compared to a traditional multiple transducer CBU system. Secondarily, we sought to determine whether diagnostic quality images could be obtained from the two comparison ultrasound systems.

## METHODS

### Study Design

This was a prospective, randomized, crossover feasibility study. The hospital’s institutional review board approved the research study. The manufacturers of the CBU or the HCU had no input into the study design, data collection, data analysis, or manuscript preparation.

**Population Health Research Capsule**

What do we already know about this issue?

*Task switching by selecting different transducers during the ultrasound evaluation of patients with undifferentiated hypotension (UHP) may lead to diagnostic and therapeutic delays.*

What was the research question?

*The primary aim was to measure the time to perform a UHP with a single- compared to a multiple-transducer ultrasound system.*

What was the major finding of the study?

*The UHP was performed significantly faster with a single transducer hand carried device compared to a cart-based system.*

How does this improve population health?

*In time-critical ultrasound studies, time savings associated with using a single transducer hand carried device may positively impact patient outcomes.*

### Study Setting and Population

The study was conducted over a two-month period of time on a convenience sample of ED patients. We conducted the study in an urban, academic ED with an annual census of approximately 60,000 visits. Patients ≥ 18 years of age and with systolic blood pressure readings ≥ 100 millimeters mercury and no signs of inadequate perfusion were evaluated for further eligibility in the study. This approach was taken for this pilot study because performing two sequential UHP on patients who were hypotensive was determined to place patients at unnecessary risk by delaying care. Exclusion criteria were one or more of the following: inability of the patient to tolerate the positioning for the ultrasound examination; traumatic mechanism of injury; or all views of the UHP could not be obtained. After obtaining written informed consent to participate in this study, each patient underwent a brief ultrasound examination of their heart and abdominal organs with a CBU by one of the study investigators to determine the feasibility of acquiring the required views of a complete UHP.

### Study Protocol

#### Ultrasound Measurements

Ultrasound studies were performed by a convenience sample of EPs with extensive experience performing an UHP. Participants included three ultrasound faculty, four ultrasound fellows, and one postgraduate year-4 emergency medicine resident. Written informed consent to participate in this study was obtained from each of the physicians. The participants were instructed to perform two UHPs on each enrolled patient. They were randomly assigned to perform the first examination with either the single-transducer HCU or the multi-transducer CBU. The second examination was performed with the device not selected for the first examination. The second examination was performed immediately after the first was completed. The HCU examinations were performed with the Butterfly iQ device, and the CBU examinations were completed with the GE LOGIQ S7 Expert (GE Healthcare, Chicago, IL) using the 3 SP-D (1.6–5.5 MHz), C1-5-D (1.8–5.0 MHz), and ML5-15 (5.0–15 MHz) transducers. The CBU incorporates a triple transducer connector; so switching between different transducers occurs by selecting a button on the keypad without having to detach or reattach different transducers.

Ultrasound hypotension protocols were performed following strict guidelines for the order of acquiring images: cardiac; IVC; abdominal focused assessment with sonography in trauma (FAST) exam; abdominal aorta; and lung, using a previously described protocol.^6^ Emergency sonographers were instructed to obtain specific views that included parasternal long axis and subxiphoid of the heart and IVC at the influx of the hepatic veins with a cardiac preset and the most appropriate transducer. Participants were then instructed to switch to an abdominal preset and the most appropriate transducer to perform the abdominal components of the FAST exam, which included the hepatorenal region to visualize the inferior aspect of the liver, Morison’s pouch, right kidney, and subdiaphragmatic space; the splenorenal region to visualize the inferior aspect of the spleen, splenorenal recess and the subdiaphragmatic space; and transverse and longitudinal views of the bladder. The emergency sonographer was then instructed to switch to an abdominal vascular or aorta preset and the most appropriate transducer to scan the abdominal aorta from the superior mesenteric artery inferiorly to the iliac bifurcation in transverse and longitudinal planes. Lastly, the emergency sonographer was instructed to select a lung preset and the most appropriate transducer to scan the left and right anterior pleural lines. The emergency sonographers completed the UHP in the same order and using the same presets with the HCU and CBU devices. They were blinded to the hypothesis of the study.

To simulate access to the different types of ultrasound devices, the HCU accompanied the emergency sonographer into the patient’s room. The CBU was plugged in, turned on, and placed immediately outside of the patient’s room.

Time measurements were collected by an independent observer using a stopwatch. For the CBU, the stopwatch was started when the emergency sonographer was instructed to perform the UHP and included the time to transport the device into the patient’s room, plug it in, and acquire each view of the UHP. A battery powers the CBU when it is not plugged in; so no time was devoted to shutting down and turning on the machine. Additionally, time required for task switching between the different presets and transducers (cardiac to abdomen, abdomen to aorta, aorta to linear) was also recorded. For the HCU, the stopwatch was started when the emergency sonographer was instructed to perform the UHP, but it did not include transport time since the device was already in the patient’s room. All of the time measurements were taken in an identical fashion for the HCU and the CBU systems. After each view of the UHP was obtained, the emergency sonographer was asked whether the image was “diagnostic quality” as a “yes” or “no” response and the independent observer recorded their response. “Diagnostic quality” was defined as the ability of the emergency sonographer to visualize anatomy and landmarks to determine the presence or absence of pathology in a particular view.

### Data Management and Statistical Analyses

All data were entered into an electronic spreadsheet (Microsoft Excel, Microsoft Corporation, Redmond, WA), transferred into native SAS format, and all statistical analyses were performed using SAS Version 9.4 (SAS Institute Inc, Cary, NC). Descriptive statistics are reported, including means and standard deviations (SD), medians with interquartile ranges (IQR), and percentages with 95% confidence intervals (CI). We made group comparisons using absolute differences, precision estimates (ie, 95% CIs), and bivariate statistical tests (ie, Wilcoxon rank sum for continuous data and Fisher’s exact test for categorical data), while accounting for correlation from the crossover design. A *P*-value <0.05 was considered statistically significant, and no adjustments were made for multiple comparisons.

## RESULTS

We approached 31 patients for enrollment. Two patients were excluded after the pre-scan due to the inability to obtain adequate views for the entire UHP. Twenty-nine patients were scanned by one of eight EPs. Each EP scanned at least three but no more than four patients. We included in the analysis 20 male patients aged 29–74 years and nine female patients aged 22–71 years. When comparing the HCU to the CBU device, the median time to complete the UHP was 4.3 vs 8.5 minutes (*P* <0.0001), respectively (Table 1). When the transport and plugin time were excluded, the median times were 4.1 vs 5.8 minutes (*P* <0.0001), respectively. There was no significant difference in the number of images judged by the emergency sonographer to be diagnostic quality obtained by the two devices, although there was a slight increase in the number of diagnostic-quality images from the parasternal long axis cardiac and IVC views with the CBU device and the four-chamber cardiac and aorta views with the HCU device (Table 2). Three points of task switching were identified, and the transitions between cardiac to abdomen and aorta to lung were significantly quicker with the single transducer HCU compared to the multi-transducer CBU device (Table 3).

## DISCUSSION

The objective of this study was to compare the time to complete an UHP using two different types of ultrasound devices. Our results demonstrated that using a single transducer HCU allows emergency sonographers to complete an UHP significantly faster than with a multi-transducer CBU system and both systems delivered adequate imagery to render a diagnosis as assessed by expert sonographers. To our knowledge, this is the first study to compare the time for image acquisition and task switching between different types of ultrasound devices, one that uses CMUT technology vs a traditional, piezoelectric transducer CBU system.

Prior study has focused on the time needed to complete certain time-sensitive, point-of-care ultrasound (POCUS) examinations,^8^ but only one study has reported the time required to complete an UHP, which on average was 5.8 ±2.1 minutes.^2^ Interestingly, in our study the median time to complete an UHP using a CBU device was also 5.8 minutes (4.73–7.67) after excluding set-up time. In comparison, examinations completed with the single-transducer HCU took 4.08 (3.43–5.35) minutes. While the time to acquire images likely has a negligible effect on a patient’s clinical course and outcome for the majority of POCUS examinations, the UHP is an exception. Commonly described etiologies of undifferentiated hypotension include significant dehydration, pericardial effusion, severe left ventricular dysfunction, free peritoneal fluid, and abdominal aortic aneurysm,^2^ which are extremely time-sensitive conditions requiring prompt diagnosis and intervention. While our study did not assess clinical outcomes, it can be argued that the significant times savings associated with using a single-transducer HCU will improve outcomes in patients with these types of critical presentations, which is an area of potential future study.

Task switching is defined as suspending a primary task to attend to a secondary task. It is a common occurrence in emergency medicine that has been associated with decreased efficiency.^9^ Multiorgan ultrasound examinations are a form of task switching since the emergency sonographer interrupts their scanning protocol to switch transducers and presets for the next component of the examination. A number of factors contribute to the significance of this type of task switching, such as a user’s familiarity with the ultrasound equipment, whether the transducer is changed manually or electronically, or the complexity of selecting presets.

The CBU device in this study uses a touch-screen interface to select different transducers and to separately select the imaging preset. The HCU device uses a single transducer that is attached to an iPhone. Preset selection is controlled with a single, pull-down menu on the iPhone. The device is configured to associate specific imaging parameters with each preset. For example, the cardiac preset configures the device to image like a phased array transducer; the abdominal or FAST preset images similarly to a curved transducer; and the lung preset is similar to a linear high-frequency transducer. Not surprisingly, the time devoted to task switching was significantly less with the single-transducer HCU compared to the multi-transducer CBU device (Table 3). While time is one metric to evaluate the impact of task switching, research has shown that task switching has additional effects, such as mental delay, prolonged duration of activity, reduced quality, and increased workload.^10^ While we only addressed time in our study, future research might address additional factors related to task switching.

Access to ultrasound equipment is a common issue in the point-of-care setting.^11^ Frequent barriers include the number of machines in a clinical area, the geography of where ultrasound machines are stored relative to where they are used, or the need to perform an ultrasound examination while a machine is in use with another patient. We attempted to simulate access an emergency sonographer would encounter with the different ultrasound devices by placing them in specific locations before the UHP was started. While the CBU device was placed outside the patient’s room, it was turned on and plugged in, so it was readily accessible, especially compared to many POCUS environments where access is more limited. The median time savings for each examination was over four minutes, which suggests that access to HCU devices has the potential to significantly improve the efficiency of emergency sonographers. This effect will likely be magnified if multiple POCUS examinations are performed with a HCU during the context of a clinical shift. For instance, if an EP performs five POCUS examinations during a clinical shift, immediate access to a single-transducer HCU device could result in 20 minutes of overall time saved.

The ability to acquire diagnostic-quality images is an extremely important feature when comparing different ultrasound devices. Prior studies have found no significant difference in the diagnostic quality and the ability of bedside sonographers to interpret images from a HCU device compared to a CBU.^12^–^15^ Our findings were similar as no specific view was associated with a significant difference between the HCU and the CBU and the number of images the emergency sonographer deemed to be of diagnostic quality. This data should be interpreted with caution, since only the ability to acquire images of diagnostic quality was assessed and not the presence or absence of pathology. Additionally, we made no direct comparison in image quality between the HCU and the CBU. Future study will need to compare the ability of a single-transducer HCU to detect and exclude pathology compared to standard POCUS systems.

## LIMITATIONS

While we enrolled patients in a clinical setting, this was a feasibility study; thus, none of the patients were hypotensive. Therefore, it is unclear whether the time-savings between the HCU and CBU systems would be maintained in a clinical situation when the patient is in shock. Additionally, none of the patients had pathology that would typically be assessed with an UHP. We also enrolled patients when the study investigators and emergency sonographers were available; thus, the enrolled patients were a convenience sample that may have introduced selection bias into our study population. We only compared one HCU device (Butterfly iQ) and one CBU system (GE LOGIQ S7 Expert). It is possible that time savings would be different if alternative ultrasound machines were studied.

We also performed a pre-scan on all patients. While this approach screened out patients with difficult anatomy, it was performed with the CBU device to ensure that all views of the UHP could be obtained. And while we mandated specific presets and transducers be used for the different aspects of the UHP, there is a possibility that time savings can occur if all views are obtained without changing a preset and using a single transducer, such as a curvilinear. We also asked emergency sonographers to self-determine the diagnostic quality of the ultrasound images from the two different ultrasound systems; therefore, it is possible that quality differences existed that an independent review would have detected.

Additionally, because no images or clips were saved, independent, retrospective review of the images was not possible. Images were not stored because the Butterfly iQ was not integrated into the ordering and archival process at our institution; therefore, the comparison between the HCU and CBU would not have been equivalent. We also did not require labels or text to be added to the images, which may have affected the time to complete studies on either a CBU or HCU; however, it can be argued that labels are not a necessary component of scans performed on hypotensive patients.

## CONCLUSION

We found that UHP examinations were performed significantly faster using the single-transducer, HCU device compared to a traditional multiple-transducer CBU system with no difference between the HCU and the CBU and the number of images the emergency sonographer deemed to be of diagnostic quality. In time-critical ultrasound studies, such as the UHP examination, time savings associated with using CMUT transducers may positively impact patient outcomes. For future studies, we recommend comparing ultrasound systems equipped with CMUT technology vs standard CBUs in patients with undifferentiated hypotension and assessing image quality along with time to diagnosis or change in management.

## Figures and Tables

**Table 1:** Total time to complete ultrasound hypotension protocol, using handheld Butterfly vs cart-based GE devices.

Time	Butterfly	GE	Difference	95% CI	P-value
Total time 1*					
Mean, (SD)	5.55 (3.51)	9.57 (3.73)	−4.02	(−5.93 – −2.12)	<0.0001
Median, (IQR)	4.28 (3.63 – 5.62)	8.52 (7.17 – 9.57)	−4.23	(−5.48 – −2.98)	<0.0001
Total time 2†					
Mean, (SD)	5.32 (3.54)	7.13 (3.51)	−1.81	(−3.67 – 0.04)	0.0556
Median, (IQR)	4.08 (3.43 – 5.35)	5.8 (4.73 – 7.67)	−1.72	(−2.78 – −0.65)	<0.0001

* Total time (minutes) includes transport and plug-in.

† Total time (minutes) excludes transport and plug-in.

*UHP*, ultrasound hypotension protocols; *CI*, confidence interval; *SD*, standard deviation; *IQR*, interquartile range.

**Table 2:** Number of diagnostic-quality images, handheld Butterfly vs cart-based GE ultrasound device.

Component of UHP exam	Butterfly N = 29 N (%, 95% CI)	GE N = 29 N (%, 95% CI)	Absolute difference 95% CI	P-value^*^
PLAX	27 (93, 77 – 99)	28 (97, 82 – 100)	−3 (−15 – 8)	1.00
4C View	26 (90, 73 – 98)	25 (86, 68 – 96)	3 (−13 – 20)	1.00
IVC	23 (79, 60 – 92)	24 (82, 64 – 94)	−3 (−23 – 17)	1.00
RUQ	29 (100, 88 – 100)	29 (100, 88 – 100)	0 (0 – 0)	–
LUQ	29 (100, 88 – 100)	29 (100, 88 – 100)	0 (0 – 0)	–
Bladder	29 (100, 88 – 100)	29 (100, 88 – 100)	0 (0 – 0)	–
Aorta	25 (86, 68 – 96)	23 (79, 68 – 96)	7 (−12 – 26)	0.73
RL	29 (100, 88 – 100)	29 (100, 88 – 100)	0 (0 – 0)	–
LL	29 (100, 88 – 100)	29 (100, 88 – 100)	0 (0 – 0)	–

*UHP*, ultrasound hypotension protocols; *CI*, confidence interval; *PLAX*, parasternal long axis; *4C*, four chamber; *IVC*, inferior vena cava; *RUQ*, right upper quadrant; *LUQ*, left upper quadrant; *RL*, right leg; *LL*, left leg.

**Table 3:** Comparison of task-switching time, handheld Butterfly vs GE cart-based ultrasound device.

Task switching	Butterfly	GE	Difference	95% CI	P-value
Total time 1					
Mean, (SD)	5.55 (3.51)	9.57 (3.73)	−4.02	(−5.93 – −2.12)	<0.0001
Median, (IQR)	4.28 (3.63 – 5.62)	8.52 (7.17 – 9.57)	−4.23	(−5.48 – −2.98)	<0.0001
Total time 2					
Mean, (SD)	5.32 (3.54)	7.13 (3.51)	−1.81	(−3.67 – 0.04)	0.0556
Median, (IQR)	4.08 (3.43 – 5.35)	5.8 (4.73 – 7.67)	−1.72	(−2.78 – −0.65)	<0.0001
Switching 1					
Mean, (SD)	0.13 (0.06)	0.30 (0.08)	−0.17	(−0.21 – −0.13)	<0.0001
Median, (IQR)	0.13 (0.08 – 0.17)	0.30 (0.23 – 0.35)	−0.17	−0.22 – −0.12	<0.0001
Switching 2					
Mean, (SD)	0.09 (0.08)	0.08 (0.10)	0.01	(−0.04 – 0.06)	0.6893
Median, (IQR)	0.08 (0.00 – 0.13)	0.00 (0.0 – 0.2)	0.08	(−0.02 – 0.18)	0.0825
Switching 3					
Mean, (SD)	0.12 (0.05)	0.33 (0.23)	−0.20	(−0.30 – −0.11)	<0.0001
Median, (IQR)	0.10 (0.10 – 0.13)	0.28 (0.23 – 0.38)	−0.18	(−0.23 – −0.14)	<0.0001
Total switching					
Mean, (SD)	0.34 (0.14)	0.71 (0.31)	−0.37	(−0.37 – −0.50)	<0.0001
Median, (IQR)	0.33 (0.23 – 0.42)	0.67 (0.48 – 0.82)	−0.33	(−0.45 – −0.21)	<0.0001

Total time 1 (minutes): includes transport and plug-in.

Total time 2 (minutes): excludes transport and plug-in.

Switching 1 (minutes): cardiac to abdomen preset.

Switching 2 (minutes): abdomen to abdomen deep preset.

Switching 3 (minutes): abdomen deep to lung preset.

*CI*, confidence interval; *SD*, standard deviation; *IQR*, interquartile range.
